# Criterion-Referenced Sex-Specific Six-Minute Walk Distance Cut-Offs for Staging Alzheimer’s Disease

**DOI:** 10.3390/jcm15145374

**Published:** 2026-07-09

**Authors:** Ines Ben Ayed, Emna Makni, Achraf Ammar, Mehdi Ben Brahim, Mohamed Elloumi

**Affiliations:** 1 Research Laboratory, Exercise Physiology and Physiopathology: From Integrated to Molecular “Biology, Medicine and Health”, LR19ES09, Faculty of Medicine of Sousse, Sousse University, Sousse 4000, Tunisia; ines.benayed@isseps.usf.tn (I.B.A.); emakni08@gmail.com (E.M.); 2 Laboratory of Human and Artificial Cognition (EA 4004), Psychology UFR, University of Vincennes/Saint-Denis, 93200 Saint-Denis, France; 3 Research Laboratory, Education, Motricity, Sport and Health (EM2S), LR15JS01, High Institute of Sport and Physical Education of Sfax, University of Sfax, Sfax 3000, Tunisia; 4 Department of Training and Movement Science, Institute of Sport Science, Johannes Gutenberg University Mainz, 55122 Mainz, Germany; 5 Research Laboratory, Molecular Bases of Human Pathology, LR19ES13, Faculty of Medicine of Sfax, University of Sfax, Sfax 3000, Tunisia; 6 Department of Nutrition and Food Technology, School of Agriculture, The University of Jordan, Amman 11942, Jordan; 7 Sport Sciences and Diagnostics Research Group, GS-HPE Department, Prince Sultan University, Riyadh 11586, Saudi Arabia; mbrahim@psu.edu.sa

**Keywords:** Alzheimer’s disease, 6MWT, functional capacity, cut-off points, AD severity, mobility assessment

## Abstract

**Background:** Functional decline emerges early in Alzheimer’s disease (AD) and may support clinical staging. However, criterion-referenced thresholds for interpreting the six-minute walk distance (6MWD) across AD stages are lacking. This study aims to derive sex-specific 6MWD cut-off values to differentiate mild cognitive impairment (MCI) from moderate AD dementia. **Methods:** In this cross-sectional study, 233 community-dwelling adults (128 women) were consecutively recruited from a neurology department and classified using IWG-2 criteria (MCI: MMSE ≥ 26; moderate AD dementia: MMSE 10–19). All participants completed a standardized 6 min walk test (6MWT) following American Thoracic Society guidelines. Receiver operating characteristic (ROC) analyses were performed overall and by sex, optimal thresholds were selected using Youden’s index, and diagnostic indices such as area under the curve (AUC), sensitivity, specificity, 95% confidence intervals (CI), likelihood ratios (LR) and odds ratio (OR) were computed. **Results:** Overall, participants with moderate AD dementia exhibited substantially lower 6MWD values than those with MCI (313.7 ± 46.5 m vs. 461.2 ± 60.1 m, *p* < 0.001). The optimal overall threshold was 390 m, yielding an AUC of 0.97, sensitivity of 98.0%, and specificity of 79.4%. Sex-specific thresholds were 389 m in men (AUC = 0.96, sensitivity = 96.4%, specificity = 79.3%) and 367 m in women (AUC = 0.98, sensitivity = 97.8%, specificity = 87.7%). **Conclusions:** The 6MWD demonstrates strong discriminatory ability between MCI and moderate AD dementia in this sample. Sex-specific thresholds may support functional staging and monitoring but require internal and external validation before clinical implementation.

## 1. Introduction

Alzheimer’s disease (AD) is a major cause of disability and loss of independence in older adults worldwide and represents a growing public health challenge [1]. Although AD is traditionally defined by progressive cognitive decline, converging evidence increasingly conceptualizes it as a multisystem disorder in which impairments in mobility and functional exercise capacity emerge early and contribute substantially to adverse outcomes, including accelerated clinical progression and increased risk of institutionalization [2,3,4]. Functional impairments, including reductions in gait performance and walking endurance, reflect the integrated functioning of neural, cardiometabolic, and musculoskeletal systems and therefore provide clinically relevant information that complements traditional cognitive assessments [3,4].

Among feasible functional assessments, the six-minute walk test (6MWT) offers a simple, low-cost, and reproducible measure of functional exercise capacity with strong prognostic value in older adults and clinical populations [5,6]. The six-minute walk distance (6MWD) reflects habitual mobility performance under sustained effort and has been consistently associated with disability, hospitalization, survival, and quality of life [7]. Importantly, in individuals with mild cognitive impairment (MCI), lower 6MWD has been linked to reduced independence, poorer memory performance and reduced gray matter volume in brain regions relevant to neurodegeneration, supporting its sensitivity to early brain–function relationships [8,9]. These findings suggest that walking endurance may capture clinically meaningful aspects of functional decline that extend beyond purely cognitive metrics.

Although previous studies have demonstrated associations between gait-related measures, cognitive impairment, and neurostructural changes, most investigations have focused on continuous functional outcomes rather than clinically actionable thresholds [8,9]. Furthermore, gait speed and 6MWD represent complementary yet distinct dimensions of mobility. Unlike gait speed, which is typically assessed over short distances and primarily reflects instantaneous locomotor performance, the 6MWD reflects integrated functional exercise capacity requiring the coordinated interaction of cardiovascular, neuromuscular, metabolic, and cognitive systems during sustained effort [5,6,7]. Therefore, establishing criterion-referenced, sex-specific 6MWD thresholds may provide additional clinical value for functional staging within the Alzheimer’s disease continuum.

Beyond cognitive disorders, performance-based functional measures (including the 6MWT) have been widely applied across cardiovascular, metabolic, pediatric, and geriatric populations, where population-specific reference values and criterion-referenced thresholds are routinely used to improve clinical interpretation and risk stratification. For example, sex-specific handgrip strength cut-points adjusted for anthropometry have been proposed to identify cardiovascular risk in older adults [10], and prediction equations for 6MWD have been developed in obese children to enhance functional assessment accuracy [11]. Similarly, established cut-off points for gait speed and grip strength are embedded within operational definitions of frailty and cardiometabolic risk evaluation [12]. Collectively, these examples illustrate the value of criterion-referenced thresholds in translating functional performance measures into clinically meaningful tools that support risk stratification, interpretation, and individualized decision-making. Despite these advances in other domains, a notable translational gap persists regarding the use of the 6MWT in Alzheimer’s disease (AD). While normative reference values for 6MWD exist for healthy older adults and several chronic diseases, criterion-referenced, performance-based functional thresholds to complement cognitive staging across AD-related clinical phases have not been formally established. In practice, clinicians can describe 6MWD but cannot easily interpret whether a given distance corresponds to a clinically meaningful transition between stages such as MCI and AD dementia [6]. Criterion-referenced thresholds, including those derived for the 6MWD, are increasingly implemented across diverse clinical populations to improve risk stratification and inform clinical decision-making [10,11]. However, such criterion-referenced, performance-based functional thresholds remain insufficiently established in the context of cognitive decline. Applying comparable approaches to functional staging in AD may enhance the clinical interpretability of mobility impairment and support more individualized monitoring and intervention strategies [13].

This gap is particularly relevant at the transition between MCI and moderate AD dementia, a critical window during which functional decline accelerates and non-pharmacological interventions, including structured exercise programs, may still be the most actionable [13,14]. Accordingly, the present study aimed to derive sex-specific, criterion-referenced 6MWD cut-off values to differentiate individuals with MCI from those with moderate AD dementia. We hypothesized that 6MWD would demonstrate strong discriminatory accuracy and that sex-specific thresholds would improve classification performance. Establishing clinically interpretable performance thresholds may provide a practical functional marker that complements cognitive assessment and supports individualized staging and intervention planning in AD.

## 2. Materials and Methods

### 2.1. Study Design

This study employed a cross-sectional observational design aimed at establishing sex-specific, criterion-referenced cut-off values for 6MWD to discriminate between stages of AD severity.

### 2.2. Participants

An a priori sample size estimation was performed to ensure adequate precision for the primary objective of estimating the AUC for 6MWD in discriminating between MCI and moderate AD dementia. Based on previous evidence demonstrating moderate-to-strong associations between 6MWD and cognitive performance as well as structural brain markers in individuals with MCI [8,9] and considering the established reliability of the 6MWT in populations with cognitive impairment and early AD [5,6], we conservatively anticipated an AUC of approximately 0.80–0.85. To estimate an AUC of 0.85 with a two-sided 95% confidence interval (CI) width not exceeding ±0.07–0.08, assuming a case-to-control ratio of approximately 1:1, a minimum total sample size of approximately 190–210 participants was required. The final analyzed sample (n = 233, including 102 participants with moderate AD dementia and 131 with MCI) therefore provided sufficient precision to yield stable AUC estimates and reliable diagnostic performance metrics.

Participants were consecutively recruited and classified into two groups according to established clinical diagnostic criteria: MCI and moderate AD dementia. Eligibility criteria included a confirmed diagnosis, the ability to ambulate independently, and medical clearance to perform the 6MWT.

Participants were recruited from the Department of Neurology at Sahloul Hospital (Sousse, Tunisia) and were categorized into either the MCI or moderate AD dementia group prior to study enrollment based on previously established clinical diagnoses made by experienced neurologists in accordance with the International Working Group-2 (IWG-2) criteria [15]. Following inclusion in the study, all participants underwent standardized research assessments, including anthropometric measurements, physiological recordings, and the six-minute walk test (6MWT). These procedures were conducted exclusively for research purposes and were not used to establish or modify the clinical diagnosis.

Diagnostic classification was based on comprehensive clinical evaluation, including cognitive assessment using the Mini-Mental State Examination (MMSE), functional assessment using the Activities of Daily Living scale, and neuroimaging findings when available. The MMSE was administered by trained neuropsychologists using the validated French version, and assessors administering cognitive assessments were blinded to the 6MWT results. The diagnosis of moderate AD dementia was based on the IWG-2 criteria and supported by clinical evaluation and cognitive assessment. Patients classified as moderate AD dementia had MMSE scores ranging from 10 to 19, consistent with moderate cognitive impairment [15].

The diagnosis of mild cognitive impairment (MCI) was also determined according to the IWG-2 framework. Individuals in this group presented objective cognitive decline without significant loss of functional independence and scored ≥26 on the MMSE [15]. Participants with intermediate cognitive stages were not included in the present analysis in order to reduce diagnostic overlap between groups and maximize classification certainty during threshold derivation.

To ensure group comparability, inclusion criteria for both groups were: age between 65 and 75 years, living independently in a non-institutional setting, normal or corrected-to-normal vision (including color perception), and the ability to ambulate independently without assistive devices.

Exclusion criteria applied to both groups included: inability to provide written informed consent; severe cardiovascular disease (e.g., unstable angina, recent myocardial infarction, uncontrolled arrhythmia) or other medical conditions contraindicating physical activity; neurological disorders other than Alzheimer’s disease that could affect cognition or mobility (including major cerebrovascular disease identified by neuroimaging); significant musculoskeletal, orthopedic, or rheumatologic conditions impairing gait; peripheral neuropathy; acute low back or lower-limb pain; use of medications known to significantly affect exercise capacity or postural control (e.g., beta-blockers, sedatives, anticholinergics); regular structured aerobic training during the previous six months (assessed via the International Physical Activity Questionnaire-Short Form); and severe psychiatric disorders, including clinically significant depression (Geriatric Depression Scale score > 10).

All participants underwent a comprehensive clinical screening, including assessment of cardiovascular risk factors (hypertension, diabetes, dyslipidemia) and relevant comorbidities, prior to enrollment. Demographic, anthropometric, educational, and clinical data were collected for all participants. Analyses were stratified by sex to account for known physiological differences in functional performance.

### 2.3. Ethics Approval and Informed Consent

The study was conducted in accordance with the principles of the Declaration of Helsinki and complied with all applicable ethical standards for research involving human participants. The experimental protocol was reviewed and approved by the Research Ethics Committee of the Faculty of Medicine of Sousse, Tunisia (approval number: IRB 00008931).

All participants, or their legally authorized representatives when applicable, provided written informed consent prior to study participation after receiving a full explanation of the study objectives, procedures, potential risks, and benefits. Participants were informed of their right to withdraw from the study at any time without consequence. Confidentiality and anonymity of all collected data were strictly maintained throughout the study.

### 2.4. Functional Assessment

Functional exercise capacity was assessed using the 6MWT in accordance with American Thoracic Society guidelines [6]. The test was performed indoors on a 40 m straight corridor with clearly marked turning points. The six-minutes duration was measured using digital stopwatch (Accusplit EA1750XLR, Accusplit, Livermore, CA, USA). Participants walked continuously for six minutes, performing repeated turns at each end of the corridor according to standardized ATS procedures. Identical testing conditions were maintained for all participants to minimize measurement variability. Participants received standardized verbal instructions (“Walk at your own pace, covering as much distance as possible in six minutes”) and standardized encouragement at each minute (“You are doing well”, “Keep up the good work”). Participants wore comfortable footwear, and all completed the test without assistive devices, in accordance with the study eligibility criteria. Height and weight were measured using digital scale (Healthometer ProPus, Pelstar, Brideview, IL, USA) and wall-mounted stadiometer (Seca Model 213, Seca, Hamburg, Germany). Testing was scheduled in the morning (8:00–11:00 a.m.) to minimize circadian variation, and participants were asked to take their regular medications as prescribed [16]. Safety stopping rules included chest pain, severe dyspnea, dizziness, or SpO_2_ < 85%. All assessors were trained in 6MWT administration and were blinded to participants’ cognitive classification.

### 2.5. Physiological Responses

The primary outcome was the 6MWD, expressed in meters. Physiological responses were recorded using validated instruments: heart rate via polar monitor (Polar H10, Polar Electro Oy, Kempele, Finland) [17], blood pressure via automated sphygmomanometer (Omron HEM-7120, Omron Corporation, Kyoto, Japan) [18], and oxygen saturation via pulse oximeter (Masimo Radical-7, Masimo Corporation, Irvine, CA, USA) [19].

### 2.6. Statistical Analysis

Descriptive statistics were used to summarize participant characteristics and are presented as means ± standard deviations for continuous variables and frequencies with percentages for categorical variables. The normality of data distribution was assessed using the Shapiro–Wilk test.

Group comparisons between participants with MCI and moderate AD dementia were performed using independent-samples *t*-tests for normally distributed continuous variables and Mann–Whitney U tests for non-normally distributed variables. Comparisons of categorical variables were conducted using the chi-square test. Effect sizes were calculated using Cohen’s d for continuous variables to quantify the magnitude of between-group differences.

All analyses were additionally stratified by sex to account for known physiological differences in functional performance.

To determine optimal 6MWD thresholds for discriminating disease stages, receiver operating characteristic (ROC) curve analyses were performed separately for men and women. Diagnostic performance was evaluated by calculating sensitivity and specificity with 95% confidence intervals (CIs) using the Wilson score method, area under the curve (AUC) with 95% CIs, odds ratios (ORs) with 95% CIs, and positive and negative likelihood ratios (LR+ and LR−). LR+ was calculated as sensitivity/(1 − specificity), and LR− as (1 − sensitivity)/specificity. Optimal cut-off values were identified using Youden’s index, which maximizes the sum of sensitivity and specificity and is appropriate when false-positive and false-negative classifications are considered similarly consequential for functional staging support [20]. Because the objective of the present study was to derive preliminary criterion-referenced functional thresholds intended to support functional staging, internal validation procedures such as bootstrap resampling or cross-validation were not prespecified within the original analytical framework.

All statistical analyses were performed using IBM SPSS Statistics (SPSS Inc., Chicago, IL, USA, version 26.0). A two-tailed *p*-value < 0.05 was considered statistically significant.

## 3. Results

### Participant Characteristics

Table 1 presents the demographic, anthropometric, educational, and clinical characteristics of participants stratified by disease stage (MCI vs. moderate AD dementia) and sex.

Overall, participants with moderate AD dementia had significantly longer disease duration and fewer years of education than those with MCI, with large effect sizes (ES > 1.2, *p* < 0.001).

Age did not differ significantly between groups in the overall sample; however, sex-specific analyses revealed that women with moderate AD dementia were significantly older than women with MCI (ES = 1.23, *p* < 0.001). BMI was significantly higher in the moderate AD dementia group in the overall sample (ES = 0.76, *p* < 0.001), whereas height and weight showed only small and inconsistent differences across sex-stratified analyses.

Table 2 summarizes functional performance during the 6MWT and associated physiological responses.

The 6MWD was markedly lower in participants with moderate AD dementia than in those with MCI in the overall sample (313.7 ± 46.5 m vs. 461.2 ± 60.1 m; ES = −2.87; *p* < 0.001), as well as in men (333.1 ± 40.7 m vs. 481.4 ± 61.3 m; ES = −2.72; *p* < 0.001) and women (297.2 ± 45.0 m vs. 445.1 ± 54.3 m; ES = −2.97; *p* < 0.001). Resting systolic and diastolic blood pressure were significantly higher in men with moderate AD dementia than in men with MCI (both *p* < 0.001). The maximum heart rate during the test was lower in the moderate AD dementia group in the overall sample (*p* < 0.05). Differences in oxygen saturation were statistically significant in selected comparisons, with effect sizes ranging from 0.36 to 2.16.

Table 3 presents the ROC-derived cut-off values for 6MWD according to sex. In the overall sample, the optimal threshold was 390 m, yielding an AUC of 0.97 (95% CI: 0.95–0.99), sensitivity of 98.0% (95% CI: 93.4–99.6), specificity of 79.4% (95% CI: 70.1–86.7), LR+ of 4.76 (95% CI: 3.21–7.05), and LR− of 0.03 (95% CI: 0.01–0.09). In men, the optimal threshold was 389 m, with an AUC of 0.96 (95% CI: 0.93–0.99), sensitivity of 96.4% (95% CI: 87.9–99.5), specificity of 79.3% (95% CI: 64.7–89.1), LR+ of 4.66 (95% CI: 2.63–8.25), and LR− of 0.05 (95% CI: 0.01–0.17). In women, the optimal threshold was 367 m, with an AUC of 0.98 (95% CI: 0.96–1.00), sensitivity of 97.8% (95% CI: 92.3–99.7), specificity of 87.7% (95% CI: 77.2–94.1), LR+ of 7.96 (95% CI: 4.15–15.28), and LR− of 0.03 (95% CI: 0.01–0.10).

**Figure 1:**
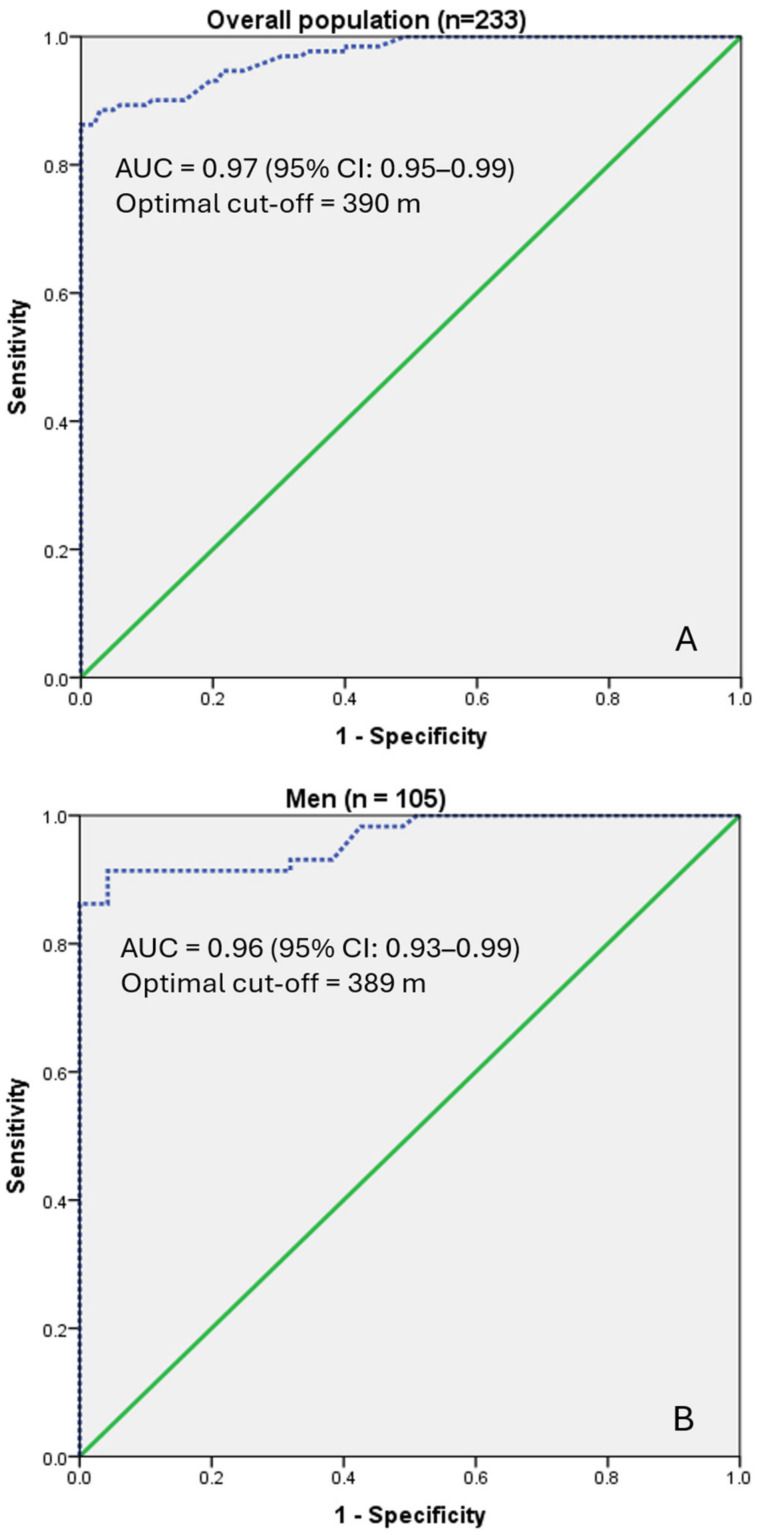

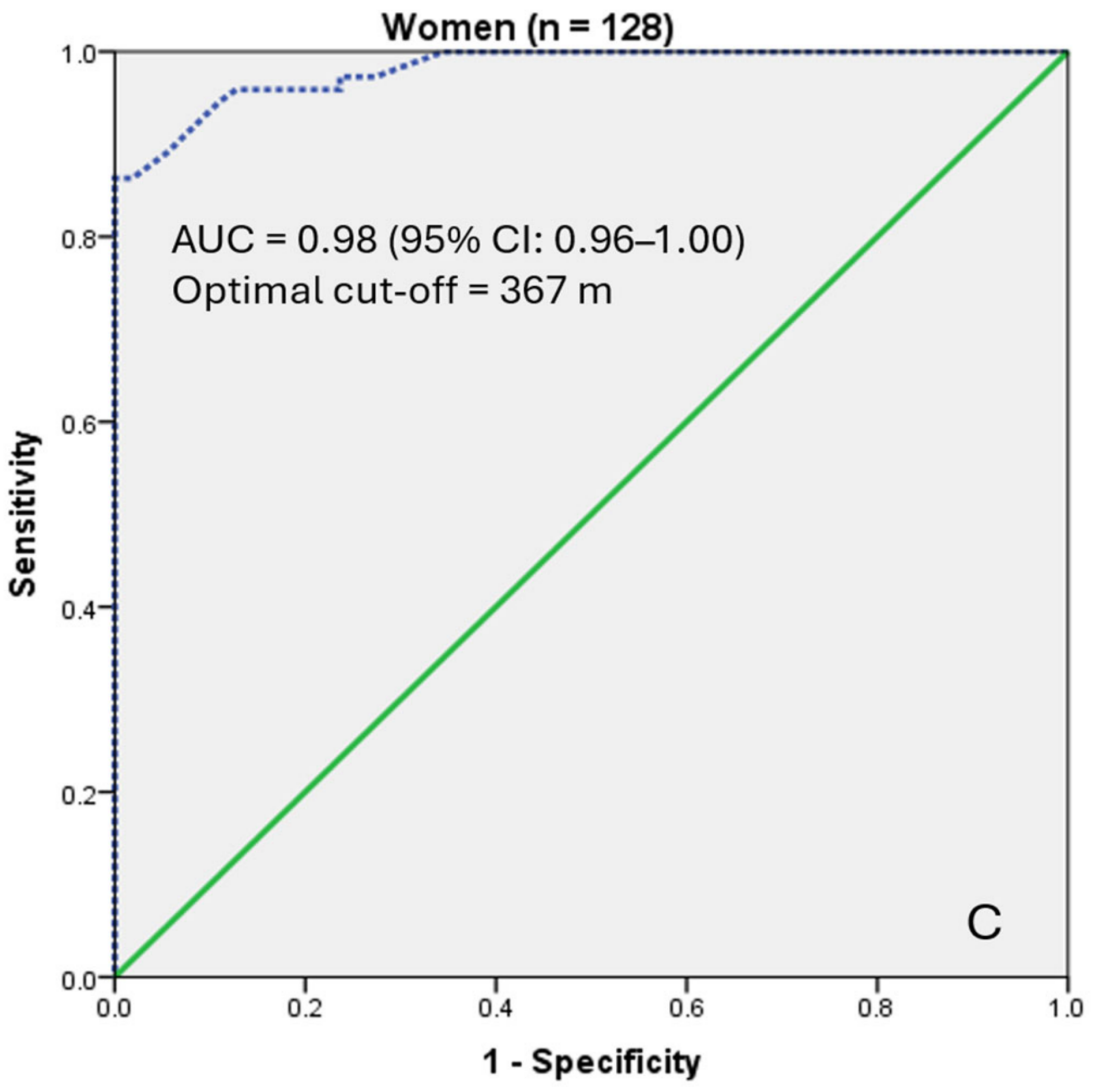
ROC curves of 6MWD for detecting AD severity in the overall sample (**A**), men (**B**), and women (**C**). ROC curves of 6MWD for detecting Alzheimer’s disease severity. Blue dashed line = 6MWD performance; green solid line = chance diagonal.

Figure 1 visually illustrates the ROC curves corresponding to the analyses reported in Table 3, including the overall sample (Figure 1A), men (Figure 1B), and women (Figure 1C).

All curves are positioned close to the upper-left corner of the ROC space, confirming excellent diagnostic performance of the 6MWD. The steep initial slope reflects high sensitivity, while the broad area under the curve highlights strong overall accuracy.

Sex-specific curves show slight differences in shape, with the female ROC curve demonstrating the highest AUC, consistent with the superior specificity and odds ratio observed in Table 3.

## 4. Discussion

The present study aimed to derive sex-specific, criterion-referenced cut-off values for 6MWD to discriminate between individuals with MCI and those with moderate AD dementia. The findings demonstrate that 6MWD is strongly associated with disease stage, with substantial between-group differences and strong discriminatory accuracy in both men and women. The large effect sizes and high AUC values indicate that walking endurance captures clinically meaningful stage-related differences in functional capacity. Previous studies have demonstrated that mobility and gait-related performance measures are sensitive to cognitive decline and may differentiate between stages of neurodegenerative disease progression [21,22]. Importantly, the derived sex-specific thresholds provide a practical and interpretable framework for translating continuous performance data into clinically relevant functional categories.

The derived sex-specific 6MWD cut-off values may be interpreted as criterion-referenced functional markers reflecting stage-related differences in AD severity. Rather than serving as standalone diagnostic tools, these thresholds may complement cognitive assessment by enhancing the clinical interpretability of mobility impairment. This integrative approach is supported by recent research indicating that motor and gait-related markers provide additional clinical information beyond cognitive testing alone [23]. Such performance-based markers could assist in functional staging and in identifying individuals who may benefit most from targeted non-pharmacological interventions, particularly structured exercise programs. Recent randomized controlled trials and systematic reviews have shown that structured physical exercise improves cognitive function and functional capacity in individuals with mild cognitive impairment [24,25].

The present findings are also consistent with broader efforts to derive criterion-referenced functional thresholds for clinically meaningful classification in other populations. For example, sex-specific handgrip strength thresholds have been proposed for cardiovascular risk stratification in older adults [26], and population-specific 6MWD equations have been developed in pediatric populations to improve functional interpretation [11]. Extending this framework to Alzheimer’s disease is clinically relevant because mobility-related markers are increasingly recognized as useful complements to cognitive assessment in identifying stage-related decline and dementia risk. In this context, the current study contributes by translating 6MWD from a continuous performance outcome into sex-specific functional cut-offs that may support staging decisions when interpreted alongside standard clinical assessment.

Several factors beyond cognitive status may also contribute to variability in 6MWD and should be considered when interpreting the proposed thresholds. Functional exercise capacity in older adults is influenced by a complex interplay of cardiovascular fitness, musculoskeletal integrity, body composition, habitual physical activity, psychological factors such as depressive symptoms and motivation, medication use, and the presence of comorbid conditions [5,6,7,8,9,10,11,12]. Moreover, 6MWD is known to be affected by demographic and anthropometric characteristics, including age, body composition, and other individual factors [27]. Consequently, the lower 6MWD observed among participants with moderate AD dementia may reflect not only disease-related cognitive and motor dysfunction but also broader health-related factors that frequently accompany advancing disease severity. In the present cohort, the higher BMI observed among participants with moderate AD dementia further highlights the potential contribution of metabolic factors to functional decline. Therefore, the proposed thresholds should be interpreted as pragmatic, unadjusted functional staging aids within comparable clinical contexts rather than disease-specific indicators independent of these influences.

Emerging evidence suggests that interventions targeting metabolic health, including nutritional optimization and modulation of the gut microbiota, may positively influence anthropometric and functional outcomes in individuals with metabolic disorders [28]. Whether similar approaches could contribute to preserving mobility and functional capacity in older adults with cognitive impairment warrants further investigation. Future studies incorporating multivariable approaches are needed to determine the extent to which 6MWD contributes unique discriminatory value beyond these potentially confounding factors, to externally validate the proposed cut-offs, and to evaluate their robustness and transportability across diverse clinical settings.

### 4.1. Baseline Characteristics and Clinical Context

The baseline characteristics provide important clinical context for interpreting the functional findings. Participants with moderate AD dementia had a markedly longer disease duration than those with MCI, which supports the internal coherence of the stage classification and is consistent with the expected progression from prodromal to more advanced clinical states. The lower educational attainment observed in the moderate AD dementia group may also be relevant in light of the cognitive reserve framework, whereby fewer years of education are associated with reduced resilience to neuropathological burden and earlier clinical manifestation of impairment [29]. The age difference observed in women, but not in the overall sample, further suggests that sex-specific trajectories of functional decline may warrant consideration. Finally, the higher BMI observed in the more severe group may reflect reduced habitual mobility and lower energy expenditure with advancing disease, although this interpretation should remain cautious given the cross-sectional design.

### 4.2. Magnitude of Functional Decline

The very large reduction in 6MWD observed in moderate AD dementia represents the central finding of the study. In practical terms, the between-group difference exceeded 145 m in the overall sample and was similarly pronounced in men and women, indicating a substantial loss of functional exercise capacity with advancing disease stage. This difference is considerably larger than established values for the minimal detectable change and the minimal clinically important difference for the 6MWT in older adults, which typically range from 30 to 50 m [30,31]. Such a magnitude strongly supports the clinical relevance of the observed functional impairment and indicates that the between-group difference cannot be attributed simply to test–retest variability or measurement error, particularly that previous work has shown that the 6MWT is a reliable measure in individuals with AD and other older-adult populations [3].

Mechanistically, this marked decline in walking endurance likely reflects the combined impact of multiple interacting factors, including motor slowing, impaired executive control, reduced dual-task capacity, lower cardiorespiratory and metabolic reserve, and diminished ability to sustain effort over time. This interpretation is biologically plausible given prior evidence linking lower 6MWD to poorer memory performance and reduced gray matter volume in older adults with MCI [8,9]. From a psychofunctional perspective, diminished 6MWD may also signify deficits in effort management, motivation, and motor–cognitive integration, without overrelying on reductionist mechanistic explanations or making unsupported causal claims [3]. Together, these considerations suggest that walking endurance in AD is not merely a peripheral motor outcome but a complex, integrative marker of brain–body health.

### 4.3. Cardiovascular and Physiological Responses

The cardiovascular findings should be interpreted cautiously but may offer additional insight into stage-related functional decline. Men with moderate AD dementia exhibited higher resting systolic and diastolic blood pressure, while the overall moderate AD dementia group showed a lower peak heart rate during the 6MWT. Although autonomic function was not directly measured, these patterns may be compatible with altered cardiovascular regulation, potentially reflecting a disruption in sympathetic–parasympathetic balance, or reduced physiological engagement during exertion. Although alterations in autonomic regulation have been reported in neurodegenerative diseases, including Alzheimer’s disease [32], autonomic function was not directly assessed in the present study. Therefore, the observed cardiovascular differences should be interpreted cautiously and may reflect multiple physiological and behavioral factors associated with disease severity rather than specific autonomic mechanisms.

This attenuated cardiovascular response may also indicate diminished central drive or effort regulation, consistent with prior research highlighting impairments in cognitive–motor integration and effort-related processes in neurodegenerative conditions [3].

By contrast, oxygen saturation remained relatively stable, suggesting that the marked reduction in walking performance is unlikely to be primarily driven by impaired pulmonary gas exchange [32]. Instead, these findings support the interpretation that limitations in 6MWD are more likely attributable to central, neuromotor, and integrative functional impairments rather than respiratory constraints per se [33]. Taken together, these observations reinforce the view that functional decline in AD reflects a complex interplay between physiological, neurological, and behavioral mechanisms.

### 4.4. Discriminatory Accuracy and Threshold Interpretation

The ROC analyses indicate strong discriminatory performance of the 6MWD for differentiating MCI from moderate AD dementia in this cohort. The AUC values of 0.96–0.98, together with LR+ values above 4 and LR− values close to 0.03, suggest that the proposed thresholds may meaningfully alter post-test probability and therefore have practical value in clinical staging. The higher specificity observed in women further supports the relevance of sex-specific thresholds rather than a single pooled cut-off. At the same time, these findings should be interpreted with caution, as the very high AUC values may partly reflect the relatively clear stage separation and sample characteristics of this single-center cohort. Accordingly, these thresholds should be regarded as promising staging-support tools rather than definitive diagnostic criteria, pending replication and validation in independent samples.

The exceptionally high AUC values should not be interpreted as evidence that walking performance alone fully captures Alzheimer’s disease severity. Rather, the 6MWD appears to reflect an important functional dimension associated with disease stage while remaining influenced by multiple physiological, behavioral, and clinical factors.

The use of Youden’s index to determine optimal cut-offs is well established in diagnostic research, as it maximizes the combined sensitivity and specificity when false-positive and false-negative classifications are considered equally consequential [20]. In this context, the reported likelihood ratios (LR+ > 4 and LR− < 0.05) further support the clinical utility of the proposed thresholds by indicating a meaningful shift in post-test probability, thereby facilitating interpretation of functional decline in clinical practice [34].

These findings are also consistent with existing literature demonstrating the feasibility and reliability of the 6MWT in AD populations and its associations with cognitive and neurostructural measures in MCI. Importantly, the present study extends prior work by translating continuous functional performance into criterion-referenced, sex-specific staging thresholds, thereby enhancing the clinical interpretability of mobility impairment within the AD continuum.

### 4.5. Strengths and Limitations

This study has several notable strengths. First, it included a relatively well-characterized clinical sample of individuals with MCI and moderate AD dementia, diagnosed according to established criteria, which enhances internal validity. Second, the use of a standardized and widely implemented functional assessment (6MWT) increases the clinical feasibility and translational relevance of the findings. Third, the derivation of sex-specific thresholds and the reporting of comprehensive diagnostic performance indices provide a structured and clinically interpretable framework for functional staging.

Nonetheless, several limitations should be acknowledged. Because of the cross-sectional design, the present findings demonstrate discriminatory ability between predefined diagnostic groups rather than longitudinal disease progression. Consequently, the proposed thresholds cannot be interpreted as indicators of transition between disease stages, predictors of future cognitive or functional decline, or markers of disease progression over time. Participants with intermediate cognitive stages were not included in the present analysis in order to reduce diagnostic overlap between groups and improve classification certainty during threshold derivation. However, this approach may have increased separation between diagnostic categories and contributed to the high discriminatory performance observed. Accordingly, the proposed thresholds should be interpreted as distinguishing MCI from moderate AD dementia rather than adjacent stages within the Alzheimer’s disease continuum, which may limit their applicability to broader clinical populations.

Although major mobility-limiting conditions were considered during participant selection, residual confounding cannot be excluded. Cardiovascular disease, musculoskeletal disorders, sarcopenia, physical activity level, depressive symptoms, medication use, and other comorbidities may have influenced 6MWD independently of cognitive status. Moreover, age, BMI, educational attainment, and other demographic and anthropometric characteristics are known to affect walking performance. Although these variables were considered descriptively, the present analyses were not adjusted for them. Therefore, future studies should determine whether 6MWD remains an independent discriminator after adjustment for these and other clinically relevant covariates using multivariable analytical approaches.

Participants were recruited from a single tertiary-care hospital, which may limit generalizability and contribute to spectrum bias. Differences in educational attainment, disease duration, cardiovascular burden, and other population characteristics may influence the stability and transportability of the proposed thresholds across populations with different ethnic, cultural, clinical, and healthcare backgrounds. Furthermore, because disease staging was primarily based on cognitive criteria, part of the observed discrimination may reflect differences in overall health status and comorbid burden rather than disease-specific mechanisms alone.

Finally, the proposed cut-off values were derived and evaluated within the same cohort and have not undergone formal internal or external validation. Internal validation procedures, such as bootstrap resampling or cross-validation, were not prespecified within the original analytical framework and therefore were not performed. Consequently, the proposed thresholds should be interpreted within the context of the present cohort and considered as preliminary and hypothesis-generating until confirmed through rigorous internal and external validation studies prior to routine clinical implementation.

### 4.6. Clinical Implications and Future Directions

From a clinical perspective, the 6MWT offers a feasible, low-cost, and scalable assessment that can be implemented in outpatient, rehabilitation, and community-based settings. The proposed sex-specific thresholds may help clinicians identify individuals whose walking endurance is disproportionately reduced relative to their cognitive classification and who may benefit from closer monitoring or targeted non-pharmacological interventions, particularly structured exercise and mobility-focused rehabilitation. This is especially relevant during the transition from MCI to moderate AD dementia, a stage at which preventive and supportive interventions may still be particularly actionable [12,14]. In addition, because exercise-based interventions have shown benefits for cognition and executive function in individuals with MCI [24,25], these thresholds may assist in identifying patients for timely supportive programs and in monitoring functional trajectories over time. Nevertheless, the proposed cut-offs should be interpreted within the broader clinical picture and should complement, rather than replace, comprehensive neuropsychological and functional evaluation.

The present findings may also provide a foundation for future longitudinal investigations. Because mobility decline often accompanies disease progression, repeated assessment of 6MWD may represent a simple and clinically meaningful marker for monitoring functional deterioration over time. Determining whether changes in 6MWD predict conversion from MCI to dementia or subsequent loss of independence may further enhance its clinical utility.

Future research should validate these sex-specific thresholds in independent cohorts and across broader clinical settings, including memory clinics, community samples, and biomarker-characterized populations. Internal validation procedures, such as bootstrap resampling or cross-validation, should also be conducted to evaluate the robustness of the proposed cut-offs. Prospective longitudinal studies are needed to determine whether these thresholds predict conversion from MCI to dementia, progression of functional dependence, falls, hospitalization, or institutionalization.

Furthermore, comparisons with complementary functional markers, including gait speed, Timed Up and Go performance, dual-task walking, and balance measures, may help clarify the role of 6MWD within multidimensional functional staging frameworks. Future studies should also evaluate whether the discriminatory value of 6MWD persists within multivariable prediction frameworks incorporating demographic, anthropometric, cardiovascular, and cognitive variables. Logistic regression approaches may provide complementary information by estimating disease-stage probabilities while assessing calibration, discrimination, and transportability across independent cohorts [21,22,23,24,25]. Nutritional factors may also contribute to mobility performance in older adults with cognitive impairment [35]. Whether strategies targeting metabolic health and mobility preservation influence functional trajectories across the AD continuum remains to be determined.

## 5. Conclusions

In conclusion, the 6MWD demonstrated strong discrimination between individuals with MCI and moderate AD dementia in this cohort, and sex-specific thresholds enhanced classification performance. These findings support the potential of 6MWD as a practical, performance-based functional staging aid in AD. However, because the proposed cut-offs were derived in a single cross-sectional sample, they should be regarded as preliminary and hypothesis-generating until confirmed through rigorous internal and external validation procedures. Future longitudinal and multicenter studies should determine whether these thresholds predict disease progression and functional outcomes and whether integrating 6MWD with complementary mobility and clinical variables improves generalizability and clinical utility.

## Figures and Tables

**Table 1:** **Basic descriptive statistics of the study participants, characterized by sex (N = 233).** Baseline characteristics of the study participants.

Variables	Overall MCI	Overall Moderate AD	Male MCI	Male Moderate AD	Female MCI	Female Moderate AD
Age (year)	67.1 ± 5.6	70.5 ± 3.6	69.3 ± 6.5	71.3 ± 3.6	65.4 ± 4.0	69.8 ± 3.5
ES/*p*-value	0.11/0.43		0.38/<0.05		1.23/<0.001	
Height (m)	1.64 ± 0.09	1.64 ± 0.08	1.69 ± 0.10	1.71 ± 0.06	1.59 ± 0.06	1.59 ± 0.05
ES/*p*-value	0.00/0.99		−0.24/0.23		0.00/0.99	

MCI: Mild Cognitive Impairment; AD: Alzheimer disease; ES: effect size.

**Table 2:** **Six-minute walk test descriptive statistics of the study participants, characterized by sex (N = 233).** Six-minute walk test performance and physiological responses.

Variables	Overall MCI	Overall Moderate AD	Male MCI	Male Moderate AD	Female MCI	Female Moderate AD
6MWD (m)	461.2 ± 60.1	313.7 ± 46.5	481.4 ± 61.3	333.1 ± 40.7	445.1 ± 54.3	297.2 ± 45.0
ES/*p*-value	−2.87/<0.001		−2.72/<0.001		−2.97/<0.001	
Resting systolic BP	121.5 ± 10.8	127.2 ± 11.4	123.6 ± 5.8	132.9 ± 9.0	119.7 ± 13.3	122.4 ± 11.0
ES/*p*-value	−0.11/0.40		1.27/<0.001		0.23/0.19	

MCI: Mild Cognitive Impairment; AD: Alzheimer disease; ES: effect size.

**Table 3:** **Receiver operating curve cut-offs for 6MWD to predict ad severity, characterized by sex (N = 233).** ROC-derived cut-off values for 6MWD according to sex.

Group	AUC (95% CI)	*p*-Value	Cut-Off	Sensitivity	Specificity	LR+	LR−	OR	Youden
Overall	0.97 (0.95–0.99)	<0.001	390	98.0	79.4	4.76	0.03	88.7	0.77
Male	0.96 (0.93–0.99)	<0.001	389	96.4	79.3	4.66	0.05	81.4	0.76
Female	0.98 (0.96–1.00)	<0.001	367	97.8	87.7	7.96	0.03	113.9	0.855

AUC: area under the curve; LR: likelihood ratios; OR: odds ratios.

## Data Availability

The data presented in this study are available on reasonable request from the corresponding author.
